# Chitosan Plus Compound 48/80: Formulation and Preliminary Evaluation as a Hepatitis B Vaccine Adjuvant

**DOI:** 10.3390/pharmaceutics11020072

**Published:** 2019-02-09

**Authors:** Dulce Bento, Sandra Jesus, Filipa Lebre, Teresa Gonçalves, Olga Borges

**Affiliations:** 1 CNC-Center for Neuroscience and Cell Biology, University of Coimbra, 3004-0504 Coimbra, Portugal; dfbento@gmail.com (D.B.); sjesus.mg@gmail.com (S.J.); filipalebre@sapo.pt (F.L.); tgoncalves@fmed.uc.pt (T.G.); 2 Faculty of Pharmacy, University of Coimbra, Pólo das Ciências da Saúde, Azinhaga de Santa Comba, 3000-548 Coimbra, Portugal; 3 Faculty of Medicine, University of Coimbra, 3004-504 Coimbra, Portugal

**Keywords:** compound 48/80, chitosan, nanoparticles, mast cell activator, vaccine adjuvant, nasal vaccination

## Abstract

Current vaccine research is mostly based on subunit antigens. Despite the better toxicity profile of these antigens they are often poorly immunogenic, so adjuvant association has been explored as a strategy to obtain a potent vaccine formulation. Recently, mast cell activators were recognized as a new class of vaccine adjuvants capable of potentiating mucosal and systemic immune responses. In this study, a co-adjuvanted delivery system was developed and characterized, combining the mast cell activator C48/80 with chitosan nanoparticles (Chi-C48/80 NPs), and the results were compared with plain chitosan nanoparticles. The adsorption of model antigens onto the NP surface as well as the biocompatibility of the system was not affected by the incorporation of C48/80 in the formulation. The stability of the nanoparticles was demonstrated by studying the variation of size and zeta potential at different times, and the ability to be internalized by antigen presenting cells was confirmed by confocal microscopy. Vaccination studies with hepatitis B surface antigen loaded Chi-C48/80 NPs validated the adjuvanticity of the delivery system, demonstrating for the first time a successful association between a mast cell activator and chitosan nanoparticles as a vaccine adjuvant for hepatitis B virus, applied to a nasal vaccination strategy.

## 1. Introduction

Traditional vaccines consisting of live attenuated or inactivated pathogens are highly immunogenic, but, due to safety concerns, development of new vaccines is being focused on the use of recombinant subunit antigens. Recombinant antigens are safer but they are often poorly immunogenic, requiring the use of adjuvants to enhance the resultant immune response. Therefore, different adjuvant approaches have been studied to enhance and/or modulate vaccine response to subunit antigens. Among them is the use of nanotechnology, a strategy that has been extensively explored and holds great promise [1,2]. Formulation of antigens in nanoparticles may offer several attractive features, namely an enhanced uptake by antigen presenting cells (APCs) [3], a depot effect with gradual release of the antigen [1,4], a cross-presentation of antigens [1,5], slower antigen processing than antigens in solution, which can result in a prolonged antigen presentation [6], and the co-delivery of antigens and adjuvants to the same cell population [2]. Besides, particulate antigens are generally more immunogenic than soluble antigens [7] and can be used to modulate the type of immune response [1]. Different nano-sized platforms such as virus-like particles, liposomes, immune stimulating complexes (ISCOMs), nanoemulsions, and polymeric nanoparticles have been explored as potential vaccine delivery systems [8,9]. Chitosan and its derivatives are among the most studied compounds for development of polymeric vaccines [10,11] because of their attractive characteristics for biomedical applications. Chitosan is a biocompatible, biodegradable, and non-toxic polysaccharide [12], obtained by deacetylation of chitin, consisting of β-(1-4)-linked d-glucosamine and *N*-acetyl-d-glucosamine monomer units. Its cationic nature, mucoadhesivity and immunostimulating properties [10] make it an attractive polymer, particularly for the design of nanoparticulate vaccines for mucosal delivery. 

Despite the potential of nanoparticles as vaccine adjuvants, it is possible to obtain a more potent adjuvant formulation by association with immunopotentiators. In fact, the concept of concomitant delivery of antigens and immunostimulatory molecules through delivery systems gained increased attention and has been appointed as a promising approach in vaccine development [2,13,14].

Mast cells (MCs), strategically located at the host surface contact with pathogens, have been recognized in recent years as important players in the development of protective immune responses [15,16,17], and the use of mast cell activators as a new class of vaccine adjuvants has begun to be explored [18]. Since then, additional studies confirmed the immunopotentiator properties of the mast cell activator compound 48/80 (C48/80) [19,20,21]. In this study, we explore the feasibility of combining the mast cell activator with chitosan nanoparticles to prepare a new adjuvant formulation for nasal vaccination. The strategy proposed here could be advantageous because, while chitosan could extend the residence time of the antigen on the nasal mucosa, MC activation could promote a local environment favorable to the development of an immune response. In particular, the application of this strategy to the hepatitis B recombinant antigen could allow the development of a nasal vaccine suitable for developing countries, where the possibility of self-administration could improve patient ease of access and compliance. Additionally, nasal vaccines are known to induce the generation of mucosal immune responses in the genital tract [22,23], which is highly attractive considering the sexual transmission of the virus. One of the biggest obstacles for vaccine development is the lack of knowledge on how to formulate complex adjuvant systems combining immunopotentiators and delivery systems [24]. Therefore, the publication of innovative methodologies for the preparation of these co-adjuvanted formulations is of utmost importance. These approaches would allow other researchers to have access to a broader range of adjuvants to test as vaccine candidates. Considering this, the present paper describes the design and characterization of a novel co-adjuvanted formulation consisting of chitosan nanoparticles associated with the mast cell activator C48/80. Stability, biocompatibility, and uptake by macrophages of the obtained nanoparticles were assessed in vitro. Finally, we tested the potential of C48/80 loaded chitosan NPs to modulate the adaptive immune response following nasal immunization using the recombinant hepatitis B surface antigen (HBsAg). To the authors’ knowledge, this was the first time C48/80 was used as a co-adjuvant for hepatitis B vaccination. 

## 2. Materials and Methods

### 2.1. Materials

A low molecular weight chitosan (deacetylation degree 95%) was purchased from Primex BioChemicals AS (Avaldsnes, Norway). Compound 48/80, bovine serum albumin (BSA), MTT (3-[4, 5-dimethylthiazol-2-yl]-2,5-diphenyl tetrazolium bromide), albumin-fluorescein isothiocyanate conjugate (FITC-BSA), trehalose, Dulbecco’s modified Eagle medium (DMEM) and Roswell Park Memorial Institute (RPMI) 1640 were obtained from Sigma-Aldrich (Sintra, Portugal). Bicinchoninic acid (BCA) assay and micro BCA kits were obtained from Pierce Chemical Company (Rockford, IL, USA). FITC was purchased to Santa Cruz Biotechnology (Santa Cruz, CA, USA). Fetal bovine serum (FBS), wheat germ agglutinin Alexa Fluor^®^ 350 Conjugate and Lysotracker^®^ Red DND 99 were obtained from Life Technologies Corporation (Paisley, UK). HBsAg was purchased from Aldevron. All other reagents used were of analytical grade. 

### 2.2. Chitosan Purification

Chitosan was purified by a method described elsewhere [25] with slight modifications. Briefly, 1 *g* of chitosan was suspended in 10 mL of a 1 M NaOH solution, and stirred for 3 h at 50 °C. The mixture was then filtered (0.45 μm membrane, MerckMillipore, Darmstadt, Germany), and the resultant pellet washed with 20 mL of deionized water. The recovered chitosan was dissolved in 200 mL of 1% (*v*/*v*) acetic acid solution and stirred for 1 h. The solution was filtered (0.45 μm membrane), and 1 M NaOH was used to adjust the filtrate to pH 8.0, resulting in purified chitosan in the form of precipitates. Purified chitosan was freeze-dried for 48 h with a Labconco freeze-dryer, model 77530 (Labconco, Kansas City, MO, USA) equipment. 

### 2.3. Characterization of the Purified Chitosan by Fourier-Transform Infrared Spectroscopy (FTIR) 

The FTIR spectra of purified and non-purified chitosan were recorded using an FTIR spectrometer (Spectrum 400, PerkinElmer, Villepinte, France) with an attenuated total reflection (ATR) top-plate accessory. The instrument operated with a resolution of 2 cm^−1^, and 30 scans were collected for each sample. Spectra were recorded between 650 and 4000 cm^−1^.

### 2.4. Nanoparticle Preparation

C48/80 loaded chitosan nanoparticles (Chi-C48/80 NPs) were prepared by adding dropwise 3 mL of an alkaline solution (5 mM NaOH) of C48/80 and Na_2_SO_4_ (0.3 mg/mL and 2.03 mg/mL, respectively) to 3 mL of a chitosan solution (1 mg/mL in acetic acid 0.1%) under high-speed vortexing. The nanoparticles were formed after further maturation for 60 min under magnetic stirring. Blank chitosan particles (Chi NPs) were obtained by preparing nanoparticles in exactly the same conditions but without C48/80. 

To evaluate the stability after freeze-drying, nanoparticles were lyophilized with 1%, 2.5%, or 5% of trehalose as cryoprotectant. All samples were lyophilized for 48 h with a Labconco freeze-dryer, model 77530 (Labconco, Kansas City, USA), at −50 °C and 100 mbar.

Chitosan–aluminum nanoparticles (Chi-Al NPs) and chitosan–poly epsilon caprolactone (Chi-PCL) NPs used in the immunogenicity study were produced as previously described by our group [26,27]

### 2.5. Characterization of Nanoparticles

#### 2.5.1. Size and Zeta Potential

Particle size was measured by dynamic light scattering (DLS) using a Delsa^TM^ Nano C (Beckman Coulter). Samples were diluted in milli-Q water and analyzed at a detection angle of 160° and a temperature of 25 °C. Zeta potential was measured by electrophoretic light scattering (ELS), after dispersion of the nanoparticles in a solution of 1 mM NaCl. 

#### 2.5.2. Morphology 

Particle morphology was evaluated by scanning electron microscopy (SEM) in a field emission scanning electron microscope (FEI Quanta 400 FEG ESEM, ThermoFisher Scientific, Waltham, MA, USA). One drop of nanoparticle suspension was mounted on a microscope stub using a double-stick carbon tape and allowed to dry overnight. Prior to image acquisition, samples were coated with gold. 

#### 2.5.3. Quantification of C48/80 Loading Efficacy

In order to evaluate the loading efficacy (LE) of C48/80, nanoparticles were centrifuged for 20 min at 8000 *g*, and C48/80 was quantified in the supernatant using a method validated by our group [28]. Briefly, 25 µL of carbonate buffer pH 9.6 was added to 175 μL of sample in a 96-well plate. Subsequently, 50 μL of a 15% acetaldehyde solution containing 1.5% of sodium nitroprusside was added and the absorbance measured at 570 nm. The loading efficacy was calculated according to Equation (1).

(1) $$C48/80\;\mathrm{LE}\;(\%)\;=\;\frac{\mathrm{total}\;C48/80\;\left(\mu g/\mathrm{mL}\right)\;-\;\mathrm{free}\;C48/80\;\mathrm{in}\;\mathrm{supernatant}\;\left(\mu g/\mathrm{mL}\right)}{\mathrm{total}\;C48/80\;\left(\mu g/\mathrm{mL}\right)}\;\times \;100$$

#### 2.5.4. FTIR Analysis

FTIR analysis of lyophilized chitosan, C48/80, Chi NPs, and Chi-C48/80 NPs was performed according to Section 2.3.

### 2.6. Stability Studies 

The short term stability of freshly prepared nanoparticle suspensions stored either at 4 °C or at room temperature (RT) was studied for a period of 15 days. Size, polydispersity index (PI), and zeta potential of three independent batches of Chi-C48/80 NPs and Chi NPs were measured. Samples were withdrawn and characterized at Days 0, 3, 5 and 15.

The stability of lyophilized Chi-C48/80 NPs was also investigated after storage for a period of 4 months at RT. At the end of the test period, samples were resuspended in milli-Q water, NPs were characterized, and the parameters were compared to the values before lyophilization. 

### 2.7. Evaluation of Loading Efficacy and Loading Capacity of Model Antigens

Loading of model antigens on a nanoparticle surface was performed by physical adsorption. Nanoparticles were centrifuged for 30 min at 4500 *g* and resuspended in acetate buffer, pH 5.7, 25 mM. Nanoparticles at a final concentration of 2.5 mg/mL were incubated with BSA, ovalbumin (OVA), or myoglobin in acetate buffer for 60 min at RT. Ratios from 7:1 to 1:1 (NP/protein) were tested for BSA, while OVA and myoglobin were incubated at a fixed weight ratio of 7:1. After incubation, particles were centrifuged at 12,000 *g* for 20 min, and the supernatant was collected. The amount of protein loaded on nanoparticles was determined indirectly by measuring the concentration of non-bound protein in the nanoparticle supernatant using the BCA or Micro-BCA protein assay (Pierce, ThermoFisher Scientific, Waltham, MA, USA) according to the manufacturer’s instructions. Loading efficacy and loading capacity (LC) were determined by Equations (2) and (3), respectively.

(2) $$\mathrm{LE}\;(\%)\;=\;\frac{\mathrm{total}\;\mathrm{amount}\;\mathrm{of}\;\mathrm{BSA}\;-\;\mathrm{non}\;\mathrm{bound}\;\mathrm{BSA}}{\mathrm{total}\;\mathrm{amount}\;\mathrm{of}\;\mathrm{BSA}}\;\times \;100$$

(3) $$\mathrm{LC}\;(\%)\;=\;\frac{\mathrm{total}\;\mathrm{amount}\;\mathrm{of}\;\mathrm{BSA}\;-\;\mathrm{non}\;\mathrm{bound}\;\mathrm{BSA}\;}{\mathrm{weight}\;\mathrm{of}\;\mathrm{nanoparticles}\;}\;\times \;100.$$

### 2.8. In Vitro Cytotoxicity Studies

Single cell suspensions of spleens from 8-week old female C57BL/6 mice (Charles River) were prepared according to a method previously described [29]. Cells were seeded in a 96-well plate at a density of 1 × 10^6^ cells/well in an RPMI 1640 medium (supplemented with 10% (*v*/*v*) fetal bovine serum, 2 mM glutamine, 1% (*v*/*v*) penicillin/streptomycin, and 20 mM HEPES) and incubated together with different concentrations of nanoparticles. After 24 h of incubation, cellular viability was assessed by MTT assay. Briefly, 20 μL of 5 mg/mL MTT in PBS, pH 7.4, was added to each well and incubated for more 4 h. The plate was centrifuged for 25 min, at 800 *g*, and the supernatants were removed. Finally, the formazan crystals produced by viable cells were solubilized with 200 µL of DMSO per well, and the optical density values were measured at 540 nm with 630 nm as a wavelength reference. The viability of non-treated cells (culture medium only) was defined as 100% and the relative cell viability calculated using Equation (4).

(4) $$\mathrm{Cell}\;\mathrm{viability}\;(\%\;)\;=\;\frac{\mathrm{OD}\;\mathrm{sample}\;\left(540\;\mathrm{nm}\right)\;-\;\mathrm{OD}\;\mathrm{sample}\;(630\;\mathrm{nm})}{\mathrm{OD}\;\mathrm{control}\;\left(540\;\mathrm{nm}\right)\;-\;\mathrm{OD}\;\mathrm{control}\;(630\;\mathrm{nm})}\;\times \;100.$$

A549 cells (American Type Culture Collection, ATCC) were seeded in a 96-well plate at a density of 10^5^ cells/mL, at 37 °C and 5% CO_2_, in a nutrient mixture F12-Ham with 10% (*v*/*v*) fetal bovine serum supplemented with 1% (*v*/*v*) penicillin/streptomycin, and incubated with different concentrations of nanoparticles. MTT assay was performed as described previously for spleen cells with minor modifications: incubation with MTT was performed over 1.5 h, and the cell supernatants were directly removed without centrifuging. 

### 2.9. Particle Uptake by Macrophages

FITC-labeled NPs were prepared by the method described in Section 2.4 using FITC-labeled chitosan. The synthesis of FITC-labeled chitosan was based on the reaction between the isothiocyanate group of FITC (Ex/Em—490/525) and the primary amino group of chitosan established by our group [30]. Briefly, chitosan was labeled by mixing 35 mL of dehydrated methanol containing 25 mg of FITC to 25 mL of a 1% (*w*/*v*) chitosan in 0.1 M acetic acid. After 3 h of reaction in the dark at RT, the FITC-labeled chitosan was precipitated with 0.2 M NaOH until pH 10. FITC-labeled chitosan was obtained by centrifugation for 30 min at 4500 *g* and the resultant pellet was washed 3 times with a mixture of methanol/water (70:30, *v*/*v*). The labeled chitosan was resuspended in 15 mL of 0.1 M acetic acid and stirred overnight. A polymer solution was dialyzed in the dark against 2.5 L of deionized water for 3 days before freeze-drying using a freeze-dry system (FreezeZone 6, Labconco, Kansas City, MO, USA).

The ability of nanoparticles to be internalized by antigen presenting cells was assessed on the mouse macrophage cell line RAW 264.7 (ECACC, Salisbury, UK). Cells were maintained in Dulbecco’s modified Eagle medium (DMEM, Sigma Aldrich, Sintra, Portugal) supplemented with 1 mM HEPES, 1 mM sodium pyruvate, and 10% of non-inactivated FBS. To evaluate the uptake of nanoparticles, RAW 264.7 cells were seeded on glass coverslips in 12-well plates at a density of 2.5 × 10^5^ cells per well and cultured at 37 °C in 5% CO_2_ overnight. On the next day, RAW 264.7 were pre-labeled with 300 nM Lysotracker^®^ Red DND 99 (Ex/Em—577/590 nm) for 30 min at 37 °C, and the culture medium was replaced by a fresh one. The cells were then incubated for 4 h with FITC-Chi NPs and FITC-Chi-C48/80 NPs at 100 µg/mL or FITC-BSA loaded nanoparticles at 50 µg/mL in DMEM.

Following uptake, cells were washed three times with phosphate-buffered saline pH 7.4 (PBS) and fixed with 4% paraformaldehyde in PBS for 15 min at 37 °C. The plasma membrane of pre-fixed cells was then labeled with 5 µg/mL of wheat germ agglutinin Alexa Fluor 350 conjugate (Ex/Em—346/442 nm) in PBS for 10 min at RT. After labeling, cells were washed twice with PBS and the coverslips mounted in microscope slides with DAKO mounting medium and examined under an inverted laser scanning confocal microscope (Zeiss LSM 510 META, Carl Zeiss, Oberkochen, Germany) equipped with imaging software (LSM 5 software, version 3.2, Carl Zeiss, Oberkochen, Germany). 

### 2.10. Immunogenicity Study 

#### 2.10.1. Nasal Vaccination

Female C57BL/6 mice of 6–8 weeks of age were purchased from Charles River (Écully, France) and housed in the Center for Neuroscience and Cell Biology (CNC) animal facility (Coimbra, Portugal) and provided food and water ad libitum. All experiments were carried out in accordance with institutional ethical guidelines and with national (Dec. No. 1005/92, 23 October 2018) and international (normative 2010/63 from EU) legislation. Groups of 5 mice were intranasally immunized on Days 0, 7, and 21 with 15 μL of vaccine formulation (7.5 μL per nostril), under slight isoflurane anesthesia. All mice, except for those in the naïve group (Group I), received 10 μg of HBsAg adsorbed onto Chi-C48/80 NPs (Group II), Chi-Al NPs (Group III), or Chi-PCL NPs (Group IV).

Samples were collected and processed as previously described [31]. Briefly, blood was collected by a submandibular lancet method on Days 21 and 42 and allowed to coagulate for 30 min prior to centrifugation at 1000 *g* for 10 min. Nasal and vaginal washes were collected on Day 42. Vaginal washes were collected by instilling 100 μL of PBS into the vaginal cavity, and the lavage fluid was flushed in and out a few times before collection. Samples were centrifuged at 11,500 *g* for 10 min, and supernatants were stored. Nasal lavage samples were collected from euthanized mice. The lower jaw of the mice was cut way and the nasal lavage collected by instilling 200 μL of sterile PBS posteriorly into the nasal cavity. Fluid exiting the nostrils was collected and spun at 11,500 *g* at 4 °C for 20 min. Collected and processed samples were stored until further analysis.

#### 2.10.2. Determination of Serum IgG, IgG1, IgG2c, and Secretory IgA

Quantification of immunoglobulins was performed using a protocol optimized by our group [27,30]. The endpoint titers presented in the results represent the antilog of the last log2 dilution, for which the OD values were at least two-fold higher than that of the naive sample, equally diluted. The log 2 end-point titers were used for statistical analysis.

### 2.11. Statistical Analysis

Statistical analysis was performed with GraphPad Prism v 5.03 (GraphPad Software Inc., La Jolla, CA, USA). Student’s t-test and ANOVA followed by Tukey’s post-test were used for two samples or multiple comparisons, respectively. A p-value <0.05 was considered statistically significant (* p < 0.05; ** p < 0.01; *** p < 0.001).

## 3. Results and Discussion

### 3.1. Purification of Chitosan 

**Figure 1:**
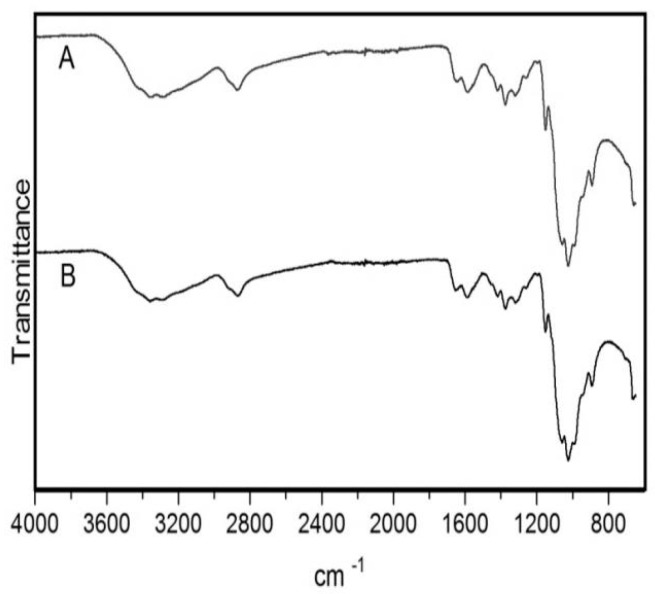
Fourier-transform infrared spectroscopy (FTIR) spectra of chitosan after and before the purification process. (A) Purified chitosan. (B) Non-purified chitosan.

Before use chitosan was submitted to a purification process to ensure the removal of any possible impurities. FTIR analysis was performed before and after the purification process to confirm the preservation of structure and integrity of the commercial polymer. The spectra obtained were in agreement with previously published data [32,33]. FTIR spectrum of chitosan showed a broad band between 3500 and 3200 cm^−1^ (Figure 1) corresponding to the stretching vibration of O–H. The peak of N–H stretching from primary amine groups was overlapped in the same region. The peak at 2869 cm^−1^ indicates C–H stretching vibrations. Peaks at 1650 and 1588 cm^−1^ correspond to C=O stretch and N–H bending, respectively. The peak at 1419 cm^−1^ belongs to the N–C stretching and the bands at 1150 and 1025 cm^−1^ are characteristic of the CO stretching vibration. No differences were observed between the spectra of non-purified and purified chitosan, which indicates that the purification process had no effect on the structure of the polymer.

### 3.2. Development and Physicochemical Characterization of C48/80-Chitosan Nanoparticles 

C48/80 loaded chitosan nanoparticles were prepared by ionotropic gelation of cationic chitosan with sulfate anions from Na_2_SO_4_. C48/80 was added to a crosslink solution and entrapped during NP formation. This method of nanoparticle preparation is extremely simple and involves mixing two aqueous solutions at RT. Despite the simplicity of the method, different conditions were tested in the laboratory before getting the final nanoparticle formulation. Based on previous data collected from our group, different concentrations of the nanoparticles components, as well as different pH and incubation conditions, were tested in order to achieve nanoparticles with the desired characteristics: submicron size, a good polydispersity, and a reasonable encapsulation of the mast cell activator C48/80. The main challenge was to associate a cationic compound, the mast cell activator C48/80, with the also positively charged chitosan. Typically, interactions with chitosan amine group are electrostatic, which favors the interaction of the polymer with anionic compounds. Consequently, the association of cationic compounds with chitosan can be trickier due to partial repulsion, since both are positively charged [34]. At the end, the preparation of chitosan nanoparticles loaded with the mast cell activator C48/80 was possible by mixing the compound with an alkalinized sodium sulfate solution prior to the preparation of the nanoparticles. 

**Figure 2:**
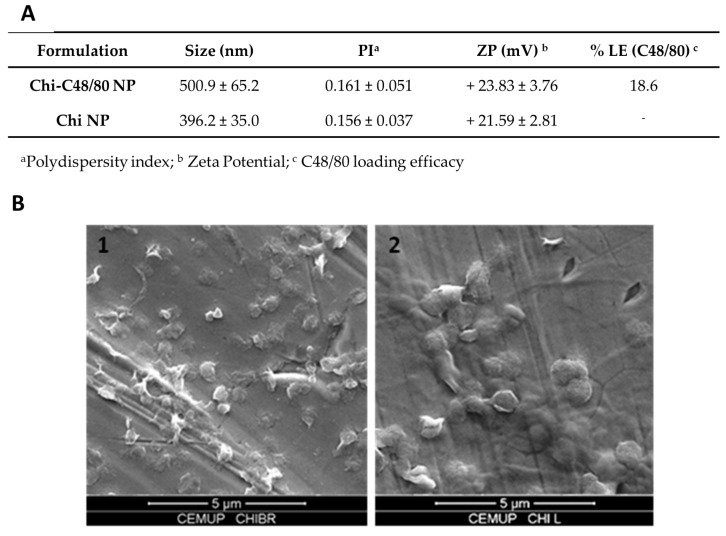
Characterization of C48/80 loaded and unloaded chitosan nanoparticles. (**A**) Size and zeta potential were measured by dynamic light scattering and electrophoretic light scattering, respectively, with a Delsa™ Nano C. C48/80 loading efficacy was measured by a colorimetric method. Mean ± SD, n ≥ 3. (**B**) SEM images of (**1**) Chi NP and (**2**) Chi-C48/80 NP. Magnification 15,000×, scale 5 µm.

Figure 2A summarizes Chi NP and Chi-C48/80 NP characteristics. The unloaded Chi NPs had an average size of 396.2 ± 35.0 nm, while the formulation loaded with the mast cell activator, Chi-C48/80 NPs, had an average size of 500.9 ± 65.15 nm. Images from scanning electron microscopy confirmed the size measured by DLS (Figure 2B1,B2). Both formulations had a narrow size distribution (PI < 0.170) and were positively charged. The incorporation of C48/80 in Chi NPs led to an increase of about 100 nm in the nanoparticle size (p < 0.001, Student’s t-test), but the nanoparticle surface charge remained unaltered. The increased mean size was a good indicator of the association of C48/80 in the nanoparticles, but the incorporation was definitively confirmed after quantification of the amount of C48/80 in the nanoparticles by a validated method [28]. The results showed that the compound was successfully incorporated into Chi NPs with a loading efficacy of 18.6%. Even if the attempts to correlate particle size and the resultant immune responses lead to conflicting findings [4,35], previous studies showed that 500 nm is in the optimal size range for uptake by APCs [36]. Therefore, not only the Chi-C48/80 NPs and Chi NPs have a suitable size for uptake, but also their positive charge is an advantage since it favors mucoadhesion, through interaction with the negatively charged sites on cell surfaces, and should also facilitate the uptake by antigen-presenting cells. 

### 3.3. FTIR Analysis of Nanoparticles

**Figure 3:**
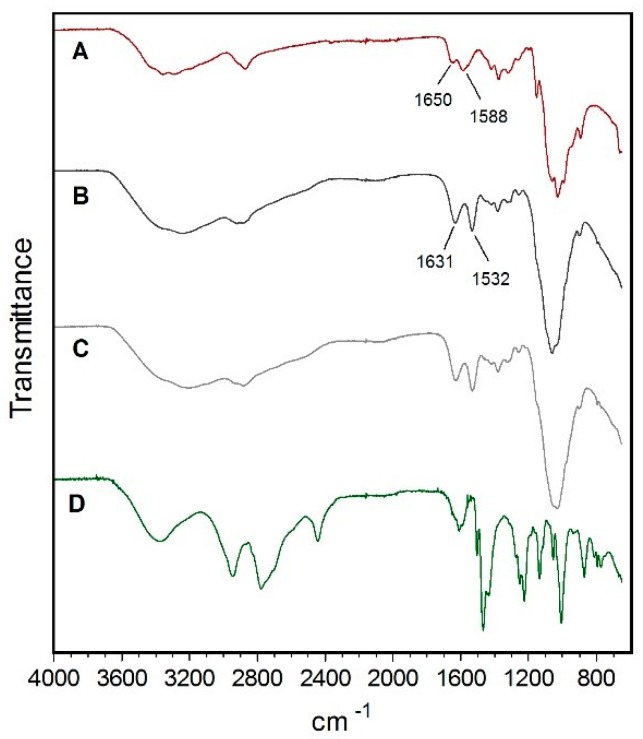
FTIR spectra of (A) Chitosan, (B) Chi NPs, (C) Chi-C48/80 NPs, and (D) C48/80.

FTIR is an important tool to analyze the interactions between groups and useful for the study of nanomaterial surface [37]. Therefore, chitosan, Chi NPs, Chi-C48/80 NPs, and C48/80 were analyzed by FTIR to characterize any potential interactions in the nanoparticles. Comparison of chitosan polymer (Figure 3A) with Chi NPs (Figure 3B) showed a shift of peaks from 1650 and 1558 cm^−1^ to 1631 and 1532 cm^−1^, respectively. This difference can be explained by the interaction between the amino groups of chitosan and sulfate ions, which resulted in the formation of the NPs by ionic crosslink [33]. The broad band between 3500 and 3200 cm^−1^ attributed to O–H and N–H bonds shifted to a lower wavelength in Chi NPs, indicating an enhancement of the hydrogen bonds interactions [38]. The same band appeared to be broader in Chi-C48/80 NPs (Figure 3C) than in Chi NPs, which also indicates enhanced hydrogen bonding in the loaded formulation [32]. No peak exclusively characteristic from C48/80 (Figure 3D) was observed on the FTIR spectrum of Chi-C48/80 NPs. This may be due to the small amount of C48/80 and therefore its chemical groups, present in the nanoparticles, which may be masked by the much higher amount of chitosan.

### 3.4. Stability Studies of the Nanoparticles 

**Figure 4:**
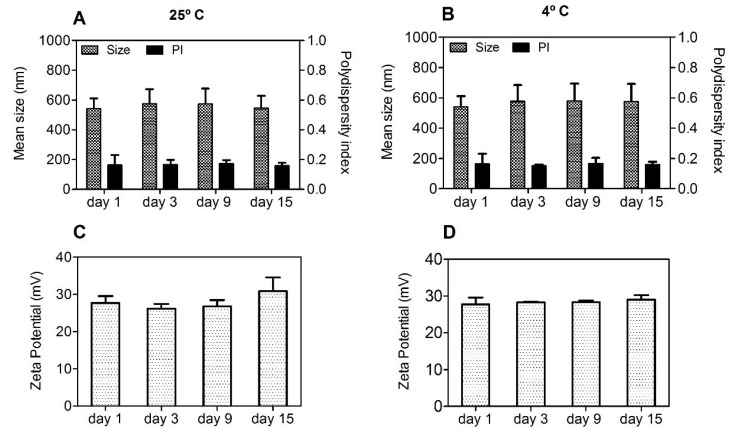
Short-term stability of Chi-C48/80 NPs at 25 °C (**A**,**C**) and 4 °C (**B**,**D**). Size, polydispersity index (PI), and zeta potential of the C48/80 loaded formulation were measured during storage up to 15 days at 25 °C or at 4 °C. Data are expressed as mean ± standard deviation (SD), n = 3.

A particle-based vaccine should be stable in relation to size throughout the process of preparation, storage, and administration. Considering this, the short-term stability of aqueous suspensions of Chi-C48/80 NPs was assessed by measuring the size, PI, and zeta potential during storage at 25 °C or at 4 °C for 15 days. Chi-C48/80 NPs showed a consistent particle size with a uniform size distribution during the test period (Figure 4A,B). Additionally, no changes in zeta potential of nanoparticles were observed (Figure 4C,D).

**Figure 5:**
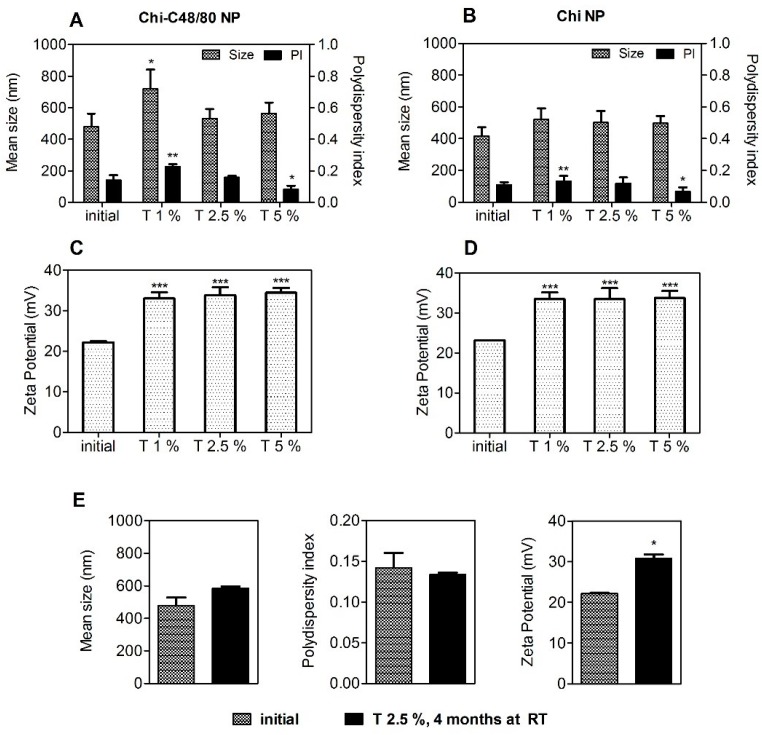
Effect of lyophilization with different concentrations of trehalose on the characteristics of nanoparticles. Size, polydispersion index, and zeta potential of Chi-C48/80 NPs (**A**,**C**) and Chi NPs (**B**,**D**) were measured before and after liophilization with 1%, 2.5%, and 5% of trehalose. (**E**) To evaluate the long-term stability of the lyophilized nanoparticles, Chi-C48/80 NPs plus 2.5% of trehalose were characterized after 4 months at room temperature (RT). Data are expressed as mean ± SD, n = 3.

The results showed that Chi-C48/80 NPs were stable up to 15 days at the tested conditions. However, it should be noted that the target function of these formulation is to act as an adjuvant for nasal vaccination after association with an antigen of interest. It is known that instability during the storage of vaccines can lead to physicochemical changes of the formulation and antigen degradation, often requiring additional steps to improve its long-term stability [4]. One effective way to guarantee this stability and to prevent antigen degradation is to lyophilize the vaccine formulation [39]. It is important that this procedure does not affect the original particle size and size distribution of the formulation since it would also influence the immune responses. Thus, the potential impact of these processes should be investigated at early stages of formulation design [4]. Considering this, we explored the feasibility of lyophilizing the developed formulations by evaluating the impact of this technique on the physicochemical properties of Chi-C48/80 NPs and Chi NPs. The preliminary data obtained revealed that freeze-drying of the developed formulations without any cryoprotectant resulted in great particle aggregation (data not shown). This destabilization of nanoparticle suspensions is very common and most likely is a result of the stress of freezing and dehydration inherent to the technique [40]. Therefore, to avoid that, in this study a fixed concentration of nanoparticles (2 mg/mL) was lyophilized with different concentrations of trehalose (1%, 2.5%, and 5% (w/v)). The physicochemical characteristics of the delivery systems were then measured after reconstitution in water and compared to the initial ones (pre-lyophilization) to see if the concentrations of cryoprotectant used were sufficient to stabilize the formulations (Figure 5).

Trehalose was selected as a cryoprotectant because it has been successfully used to preserve not only the characteristics of chitosan nanoparticles [38] but also the bioactivity of both C48/80 and an antigen [21]. The size of Chi NPs remains the same after lyophilization with all trehalose concentrations tested (Figure 5A,B). On the other hand, differences in size and PI for Chi-C48/80 NPs indicate that an adequate reconstitution was only achieved when using 2.5% or 5% of the cryoprotectant. Zeta potential of nanoparticles increased after the lyophilization with trehalose (Figure 5C,D). This can result from either a change in charge distribution on the NP surface or from the presence of trehalose on the on the NP suspension. The results suggest that 2.5% of trehalose was sufficient to achieve a successful cryopreservation of both delivery systems.

To assess if the lyophilized nanoparticles would be feasible to avoid the cold chain, Chi-C48/80 NPs lyophilized with 2.5% of trehalose were characterized after storage at RT for 4 months. Results showed that nanoparticles preserved the initial size and polydispersity for at least 4 months of storage (Figure 5E).

Overall, the results suggest that the stability of Chi NPs was not impaired by the association with C48/80. However, it is noteworthy that, even if these studies on the stability of the nanoparticles provided us with an indication about the potential of the formulations for long-term storage, they do not exclude the requirement of a more complete stability study, including antigen potency evaluation over time, for guarantee that the immunogenicity of the vaccine candidate is not affected. That would be particularly important during the development of vaccines designed to avoid the cold chain. 

### 3.5. Loading of Model Antigens

In this study, BSA, OVA, and myoglobin were used as model antigens to confirm the adsorption of proteins onto the surface of nanoparticles. The use of three different proteins with different isoelectric points (IPs) allowed us to assess the suitability of the developed delivery systems for loading different antigens of interest. The loading of proteins on nanoparticles was made by physical adsorption, a mild technique that involves simply the incubation of nanoparticles with an aqueous solution of the antigen. This approach not only helps to preserve the structure of antigen but also allows a repetitive antigen display to the APC, which mimics pathogens [1]. 

**Figure 6:**
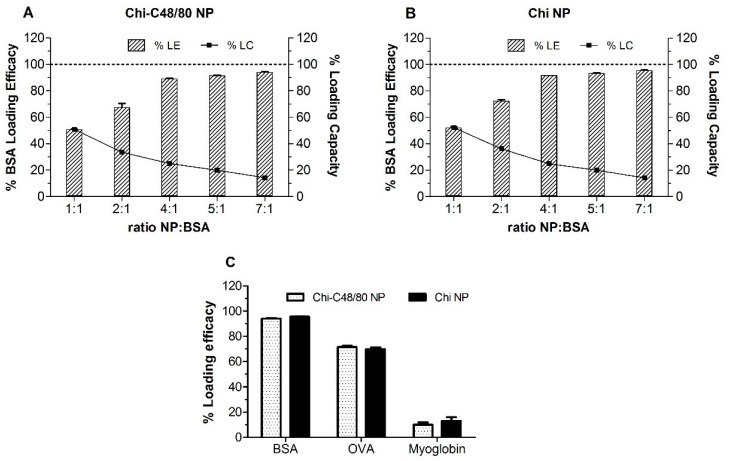
Loading of model antigens. Effect of NP/protein ratio on the protein adsorption to (**A**) Chi-C48/80 NPs and (**B**) Chi NPs. (**C**) Loading efficacy for bovine serum albumin (BSA), ovalbumin (OVA) and myoglobin at the NP/protein ratio of 7:1. Nanoparticles were incubated with different proteins for 60 min in acetate buffer, pH = 5.7 at RT. Loading efficacy (% LE) and loading capacity (% LC) were determined after quantification of unbound protein in the supernatant using the Bicinchoninic acid (BCA) assay. Bars represent mean ± SD, n = 3.

Initially different NP/BSA ratios were tested to evaluate the more efficient weight ratio of NP/protein for loading. BSA loading efficacies were very similar for both Chi-C48/80 NPs and Chi NPs (Figure 6A,B). The LE was dependent on the ratio NP/BSA: the higher the ratio, the higher the amount of protein adsorbed on NP surface, ranging from 50.8% to 94.1% and from 52.2% and 95.5%, for Chi-C48/80 NPs and Chi NPs, respectively. The LC of nanoparticles was also calculated; it represents the amount of protein that the nanoparticles are able to carry. Unlike LE, the LC of the nanoparticles decreased with the increase in the NP/BSA ratio. Loading capacity was maximum at the lower ratio tested (1:1) for both Chi NPs and Chi-C48/80 NPs. However, even if the incubation with higher amounts of protein allows the nanoparticles to carry a higher amount of the protein of interest, generally it is preferable to use the NP/protein ratio that allows the highest LE because the antigen is usually the most expensive component of the vaccine. The LE near 95%, achieved for NP/BSA = 7:1, is very favorable in formulation development since almost the entire amount of antigen used would be associated with the nanoparticles. Therefore, this ratio was selected to test the loading of OVA and myoglobin on the NP surface. 

LE of OVA and myoglobin on nanoparticles was around 70% and 10%, respectively, for both Chi-C48/80 NPs and Chi NPs (Figure 6C). These values were significantly lower than those observed for BSA. The isoelectric points of BSA, OVA, and myoglobin (4.7, 4.9, and 7.2, respectively) can help one to understand the observed results. BSA and OVA displayed high adsorption efficacies to the surface of the NPs that can be associated with the electrostatic interactions between the positively charged amino groups of chitosan and the negatively charged carboxyl groups of the proteins. On the other hand, at pH 5.7, both nanoparticles and myoglobin are positively charged, which explains the very low LE % observed for this protein. However, despite the similarity in the isoelectric points of BSA and OVA, the adsorption of these proteins was different within the same delivery system. That is because, even if the IP is helpful for predicting the loading of proteins on the NP surface, the adsorption is a complex process depending on several other factors [41]. Overall, the results suggested that the developed formulations are suitable for loading negatively charged antigens and that the adsorption of protein onto the Chi NP surface was not affected by the presence of C48/80.

### 3.6. Cytotoxicity 

**Figure 7:**
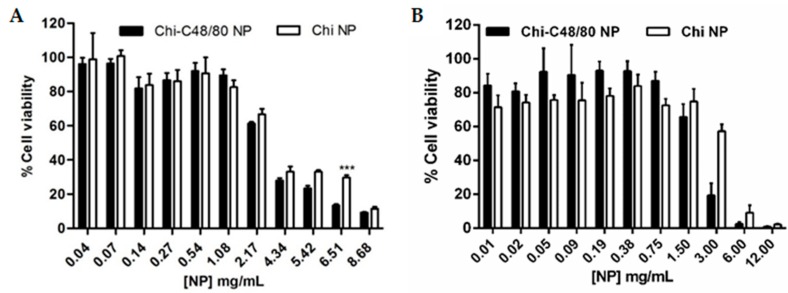
Effect of Chi-C48/80 NPs and Chi NPs on viability of spleen cells (**A**) and epithelial A549 cells (**B**). Different concentrations of nanoparticles were incubated with cells for 24 h. The cell viability was measured by MTT assay. Each result is representative of two independent experiments performed in quadruplicate for spleen cells and three independent experiments performed in quadruplicate for A549 cells (mean ± SD).

The cytotoxicity of Chi-C48/80 NPs and Chi NPs was evaluated in spleen cells and in the A549 cell line using the MTT assay. Spleen cells were chosen because they are a good representation of the different cells of the immune system and have been already used to test the toxicity of vaccine delivery systems [29,42]. Since the aim of this study was to evaluate the incorporation of a mast cell activator in a delivery system, the A549 cell line, a model of alveolar basal epithelium, was used to evaluate the potential harmful effect of the nanoparticles at the administration site. As expected, the results show that cytotoxicity was concentration-dependent. Higher concentrations of nanoparticles resulted in a decreased cell viability (Figure 7).

In spleen cells, the incorporation of C48/80 in Chi NPs did not affect the toxicity of formulations, with both formulations showing no cytotoxicity for concentrations up to 2000 µg/mL (Figure 7A). With the A549 cell line, cell viability was above 70% for concentrations up to 750 µg/mL, for both formulations (Figure 7B). However, in these cells, the presence of C48/80 in the nanoparticles reduced the cell viability more rapidly than did Chi NPs. Note that 1500 µg/mL is a very high concentration and out of the range normally used in in vitro studies. These results are in agreement with others that have demonstrated that chitosan nanoparticles are nontoxic [29,43,44].

### 3.7. Uptake Studies

**Figure 8:**
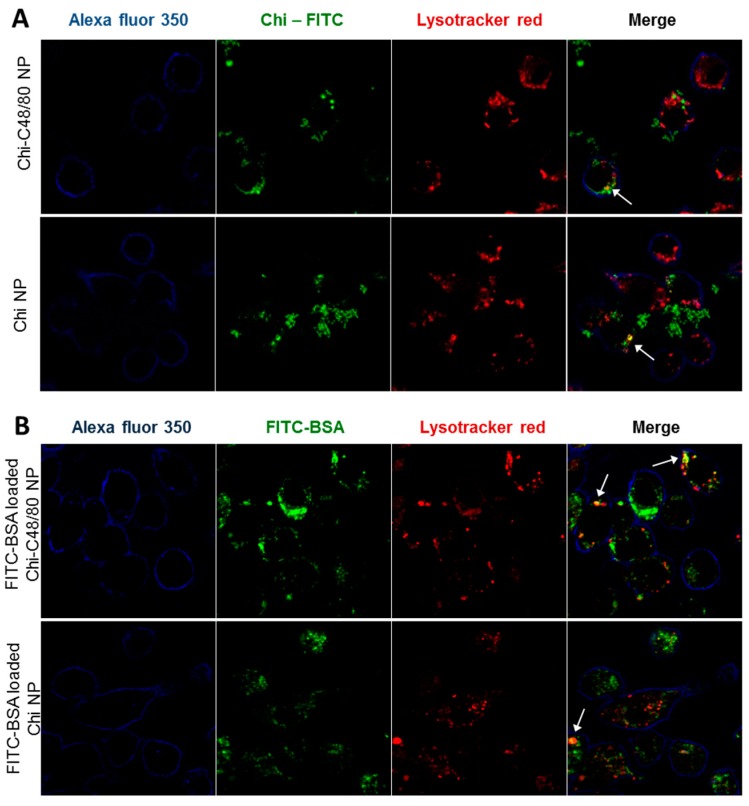
Evaluation of uptake by macrophages. (**A**) Uptake of nanoparticles was assessed by incubating 4 h, 100 µg/mL RAW 264.7 cells with Chi-C48/80 NPs or Chi NPs prepared with fluorescein isothiocyanate (FITC)-labeled chitosan (green). (**B**) Uptake of the antigen loaded on nanoparticles was evaluated by incubating the cells with FITC-BSA loaded Chi-C48/80 NPs or Chi NPs. Cells were labeled with Alexa Fluor^©^ 350 WGA (blue) to identify the membrane, and Lysotracker^©^ Red identifies the acidic endosomes and lysosomes. Arrows in the merge image show co-localization.

The uptake of nanoparticles by antigen presenting cells is favorable to an adaptive immune response [45]. Therefore, we investigated the ability of both developed formulations to be internalized by RAW 264.7 cells, a macrophage cell line widely used to explore the uptake and immune effect of vaccine delivery systems [3,7]. To visualize particle uptake by the macrophage cell line RAW 264.7, cells were incubated with FITC-labeled Chi NPs or Chi-C48/80 NPs. The intracellular location of nanoparticles was analyzed by labeling the cells with Lysotracker Red, which accumulates in the acidic endolysosomes. The results showed that the NPs were efficiently taken up by macrophages (Figure 8A). After 4 h of incubation, the FITC-NPs (green) were mostly detected on the cell cytoplasm. However, some of the compartments enclosing NPs showed acidification, as observed in the merged images between green fluorescent NPs and red fluorescent vesicles, appearing in yellow, indicating maturation of the phagolysosome (Figure 8A). To confirm that not only the nanoparticles but also the associated antigens would be internalized by antigen presenting cells, uptake studies were repeated with nanoparticles loaded with a fluorescently labeled protein. The confocal images showed an extensive internalization of FITC-BSA loaded on both Chi-C48/80 NPs and Chi NPs (Figure 8B). Similarly to what was observed with FITC-labeled NPs, the fluorescence signal of BSA was detected mostly on cell cytoplasm with only a few yellow co-localization signals observed. 

These results suggest that NPs and protein-loaded NPs might escape from the endosomes to cytoplasm which can facilitate cross-presentation and potentially mediate the MHC I antigen presentation pathway, associated with an induction of CD8+ T cell response [3]. In fact, it was demonstrated by others that chitosan-based nanoparticles could escape from endosomes [46] and that antigens delivered by Chi NPs mediate antigen presentation through both MHC I and MHC II pathways [7]. This escape mechanism and consequent cross-presentation of antigens is particularly important for the development of vaccines that require cellular immune response. 

Overall, the results showed that there were no significant differences regarding the uptake and distribution of Chi-C48/80 NPs and Chi NPs. This means that the association of the mast cell activator with the NPs not only did not impair the characteristics of nanoparticle and antigen uptake by antigen-presenting cells but also proved to be an effective antigen delivery system. 

### 3.8. Immunogenicity Study

Chi-C48/80 NPs’ ability to act as a nasal vaccine adjuvant for hepatitis B surface antigen was tested and compared with other particulate adjuvants by performing an immunogenicity study using C57BL/6 mice. The immunization schedule comprised a primary nasal immunization followed by two nasal boosts at Days 7 and 21. Therefore, in this study, we included two other established chitosan-based nano-delivery systems developed by our group, Chi-Al NPs [26] and Chi-PCL NPs [27], in order to compare adjuvanticity elicited by the new system.

**Figure 9:**
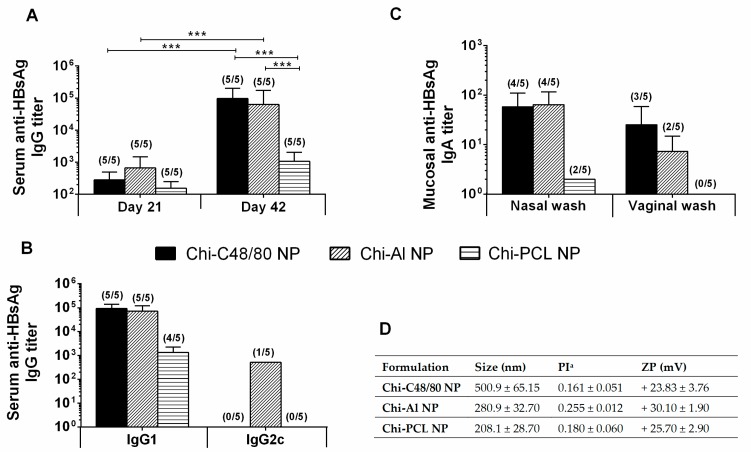
Immune profile of C57BL/6 mice after intranasal immunization. Serum anti-HBsAg IgG (**A**), IgG1 and IgG2c (**B**) and mucosal IgA (**C**) titers obtained with 10 μg HBsAg loaded into Chi-C48/80 NPs, Chi-Al NPs, or Chi-PCL NPs. Blood was collected by submandibular lancet method on Days 21 and 42, and nasal and vaginal washes were collected on Day 42. Numbers above bars represent the number of mice in which antibody levels were detected. Data (mean ± SD) correspond to responder mice of each group. Table with summary of nanoparticle size, polydispersity index, and zeta potential of the formulations used during the immunization studies (**D**).

As depicted in Figure 9A, although all formulations were able to induce detectable IgG titers at Day 21, at Day 42 Chi-C48/80 NPs and Chi-Al NPs induced significantly higher titers than Chi-PCL NPs. All delivery systems tested induced a Th2-type immune response, since the predominant IgG subclass elicited was the IgG1 (Figure 9B). Therefore, Chi-PCL NPs despite being an excellent adjuvant when administered by a subcutaneous route, was shown in this work to have a lower value when used in the intranasal route. 

The main reason to develop new and effective mucosal vaccine delivery systems, besides its ease of access and administration, is the possibility to induce mucosal antibodies at the site of entry of pathogens, actively preventing the infection. Our results showed that Chi-C48/80 NPs were able to induce mucosal anti-HBsAg IgA in the vaccinated mice (Figure 9C). Indeed, the nasal washes revealed mucosal IgA titers in 80% of the Chi-C48/80 NP-vaccinated mice, similar to Chi-Al NPs, but vaginal washes revealed 60% of responder mice in comparison to the 40% of Chi-Al NPs. All nanoparticles used in this experiment presented a positive zeta potential, characteristic of a chitosan presence (Figure 9D). Nevertheless, sizes presented a higher variation between formulations, with a Chi-C48/80 NP size about two times larger than the other nanoparticles. 

These results suggest that the adjuvant Chi-C48/80 NPs is a valuable strategy to improve the efficacy of nasal recombinant vaccines. Immunoglobulin results were slightly higher than the ones obtained with Chi-Al NPs, which adjuvanticity was already established by our group [26,30,47] and particularly for the nasal route. In fact, Lebre et al. [30] observed Chi-Al NPs complexed with pDNA from HBsAg, were able to induce humoral and mucosal antibodies against HBV. Moreover, in opposition to free HBsAg, Lebre et al. [47] also reported that HBsAg loaded Chi-Al NPs elicited HBsAg-specific IgG in nasal and vaginal washes when the vaccine was subcutaneously administered. On the other hand, when compared to Chi-PCL NPs, Chi-C48/80 NPs demonstrated a superior adjuvant activity. Chi-PCL NP adjuvanticity through subcutaneous and nasal routes was also validated by our group in a direct comparison with free HBsAg [27,48,49]. In detail, through the nasal route, free HBsAg did not induce specific IgG titers, while Chi-PLC NPs elicited a detectable immune response with an HBsAg dose 10 times lower than the control [27]. Moreover, the conclusion that Chi-C48/80 NPs are able to increase the immune response to HBsAg is reinforced when the results are compared to Chi-Al NPs and Chi-PCL NPs, since those delivery systems were already compared in vaccination studies with the commercially available HBV vaccine (Engerix^®^) [47,49]. In those studies, both delivery systems were able to induce better IgG titers than Engerix^®^ at same dose. 

Interestingly, a recent article, published by Schubert et al. [50] observed that the adjuvant activity of C48/80 for the specific immune response against ovalbumin upon intranasal immunization is independent of the MC presence. Therefore, further work aimed to elucidate the mechanistic of action of Chi-C48/80 NPs as a mucosal vaccine adjuvant would be insightful to clarify if its adjuvant activity is related with a C48/80 ability to activate MC, or if it is a mechanism similar to chitosan, that largely stems from the fact that it is a cationic polymer. In fact, chitosan nanoparticles alone are able to activate mast cells, a finding recently made by our group [51].

Overall, the association of C48/80 with chitosan NPs was found to produce a good adjuvant and thus to be a suitable approach to improving the activity of the HBsAg, through mucosal routes of administration. 

## 4. Conclusions

Chitosan is a biomaterial with appealing properties for vaccine delivery. Considering this, we designed and developed a new chitosan-based vaccine delivery system by efficiently incorporating the mast cell activator C48/80 into chitosan nanoparticles. Overall, the data obtained showed that the versatility of chitosan was associated with additional adjuvants without significantly affecting the physicochemical and biocompatible properties of the polymer. This is a characteristic of chitosan that deserves to be further explored in order to support the design of improved delivery systems or immunopotentiators to better modulate the immune response of the antigens. The delivery system was shown to have interesting features for vaccine delivery, namely, the ability to adsorb high amounts of antigen, internalization by antigen-presenting cells, and stability after lyophilization, which can be a useful future development of a cold chain-free vaccine formulation. Chi-C48/80 NPs also demonstrated adjuvant activity for the hepatitis B antigen similar to Chi-Al NPs and superior to Chi-PCL NPs, confirming its feasibility as a vaccine adjuvant through demanding but promising routes, such as the nasal route. To better design nanoparticulate immunopotentiators, it is desirable that the mechanism of the adjuvants be better studied, with the certainty that for each adjuvant we are not talking about a single mechanism, but most likely more than one that, the other way, may be competitive. This knowledge is absolutely essential, so that in the near future it will be possible to design adjuvants with more predictable immunomodulatory action.
